# Prevalence of dental caries in the first permanent molar and associated risk factors among sixth-grade students in São Tomé Island

**DOI:** 10.1186/s12903-021-01846-z

**Published:** 2021-09-28

**Authors:** Lin Que, Mao Jia, Zhen You, Li-cheng Jiang, Chun-guang Yang, Alexandre Afonso d’Oliveira Quaresma, Edgar Manuel Azevedo Agostinho das Neves

**Affiliations:** 1 State Key Laboratory of Oral Diseases, West China Hospital of Stomatology, Sichuan University, Chengdu, 610041 China; 2 Department of Biliary Surgery, West China Hospital, Sichuan University, Chengdu, 610041 China; 3 Department of Head and Neck Oncology, West China Hospital of Stomatology, Sichuan University, Chengdu, 610041 China; 4 Department of Orthodontics, West China Hospital of Stomatology, Sichuan University, Chengdu, 610041 China; 5 Department of Cardiology, The First Affiliated Hospital of Chengdu Medical College, Chengdu, 610041 China; 6 Department of Rehabilitation, Hospital of Traditional Chinese and Western Medicine of Panzhihua, Panzhihua, 617000 China; 7 Ministry of Education, Água Grand, São Tomé and Príncipe; 8 Ministry of Health, Água Grand, São Tomé and Príncipe

**Keywords:** Dental caries, Oral hygiene, Dental health for children, São Tomé and Príncipe

## Abstract

**Background:**

Dental caries is one of the most preventable oral diseases among children in developing countries. This study aims to estimate the prevalence and severity of dental caries in the first permanent molar and analyze the related risk factors among sixth-grade students in São Tomé Island.

**Methods:**

A cross-sectional study with a stratified cluster sampling method was conducted on 1855 sixth-grade school children, mainly aged 11 to 14 years old, from 10 schools in 6 regions of São Tomé Island, from April 17 to June 27, 2021. Dental caries examination was performed by using the CAST criteria (DMFT) index, and the self-administered questionnaires about family background, oral hygiene, and relevant behaviors were collected. Multivariable logistic regression was used to study risk factors related to dental caries of the first permanent molar, and all data analyses were done using SPSS version 25.

**Results:**

The prevalence of dental caries in the first permanent molar was 68.79%, without significant difference between gender, age, residence, and whether only child or not. The mean Decayed, Missing, and Filled Teeth (DMFT) index and mean Decayed, Missing, and Filled Surface (DMFS) index were 1.751 ± 1.514 and 3.542 ± 3.941, respectively. The rate of filling teeth was 5.50%, and Pit and Fissure Sealant (PFS) rate was 2.21%. The overall prevalence and DMFT index of dental caries of permanent teeth was 76.01% and 2.753 ± 4.569, respectively. The results of logistic regression analysis indicated that the frequency of candy/chocolate consumption (OR = 1.095) and fair self-assessment of dental health (OR = 1.354) were significantly associated with dental caries (*P* < 0.05).

**Conclusions:**

The high prevalence of dental caries in the first permanent molar was a public health issue among sixth-grade school children in São Tomé Island. The prevalence of dental caries, mean DMFT and DMFS scores were higher, while the rate of filling and PFS teeth were lower than the average score of other African countries. Thus, oral health education, implement oral health preaching to school children and their parents is crucial to prevent dental caries.

**Supplementary Information:**

The online version contains supplementary material available at 10.1186/s12903-021-01846-z.

## Background

Dental caries is one of the most preventable chronic and cumulative diseases, which affects 60–90% of schoolchildren as well as many adolescents and adults worldwide [1, 2]. Without proper treatment, caries can evoke oral pain, infection and eventually lead to tooth loss, affecting children’s quality of life both physically and psychologically, and becoming a global public health issue in developed and developing countries [3–5].

Dental caries is a chronic bacterial infectious disease destructing the tooth’s hard tissues by acids produced by a complex interaction, whose main factors are host susceptibility, bacteria, diet, and duration. The destruction of the tooth is a dynamic process determined by the balance between demineralization and remineralization occurring in the interface between hard tooth tissue and the surrounding environment [7–9]. Dental caries is preventable and reversible, which can be arrested at any stage, provided that protective measurements are implemented, such as fluoride application, dental care, pit and fissure sealant, and adequate health service utilization [10].

It is reported that around 1/3 of the world’s population suffers from dental caries in their permanent teeth. Over the past few decades, the global prevalence and severity of dental caries have declined in high-income countries, significant variations exist, and many children still develop caries [11]. In developed countries like the USA, caries is the most common chronic disease of childhood, which is five times more common than asthma [12]. In the UK, caries caused more than 60,000 children hospitalization for teeth removed under general anesthesia [13]. Due to the increase in sugary consumption, poor oral hygiene practices, lack of dental health policies, insufficient preventive measures, and inappropriate or unavailable dental health services, the incidence of dental caries is rapidly growing in most developing countries among children and adults [14].

Generally speaking, dental caries and their sequelae can affect children’s physical growth, self-esteem, and social development due to missing, discolored, or damaging teeth [15]. According to the statistics, the direct treatment of dental caries cost 298 billion, amounting to 4.6% of the global health expenditures [16]. The treatment of dental caries can last a lifetime, which is extremely costly, can be a significant socioeconomic burden on both individuals and health care systems.

São Tomé and Príncipe is an island country located in the Gulf of Guinea, off the western equatorial coast of Central Africa, with a population of 201,800 and 90% of which lived in São Tomé Island. However, as there has been no nationwide epidemiological data regarding the prevalence of dental caries and its associated factors, the situations of dental caries in this country remain a considerable challenge. Therefore, to reduce dental caries, the investigation of detailed information on dental caries status and related risk factors is vital for the government to develop effective policies.

The position of the first permanent molar after the eruption is relatively constant, which has the function of stabilizing the dentition, guiding the rest of the permanent teeth to an adequate position, and determining the occlusion. Its caries status can reveal the permanent dentition caries distribution of children in adulthood. According to the WHO standard, the age of 12 was considered the age of global monitoring of dental caries for international comparisons and monitoring of disease trends [17].

Within this context, this study aims to evaluate the prevalence and severity of caries in the first permanent molar among sixth-grade school children (with an average age of 11–12 years old) in São Tomé Island and to analyze related risk factors.

## Methods

### Ethical considerations

This cross-sectional study was approved by the Ethical Committee of São Tomé and Príncipe and conducted in São Tomé Island from April to June 2021.

### Study population

The target size population of this study was 6,041 sixth-grade students of 9-years-old to 15-years-old (local children normally go to school at the age of 7, which means most of the sixth-grade students were around age 12), from a total of 190 classrooms of 55 schools, according to the statistical data from the Ministry of Education of São Tomé and Príncipe (Additional file 5).

### Inclusion criteria and exclusion criteria

Inclusion criteria were: sixth-grade students attending class on the day of the survey, with informed consent obtained from each student and their parents.

Exclusion criteria were: students who were not attending class on the day of the survey were unable to cooperate with the examiner, without informed consent from each student and their parents, and students with systemic diseases or mental disorders.

### Sampling design

A stratified cluster sampling method was conducted, which was stratified by six different districts. The single population proportion formula was used to determine the sample size:

$$n = \frac{{z^{2} p\left( {1 - p} \right)}}{{e^{2} }}$$

*n* is the sample size per district; *p* is the dental caries prevalence (estimated to be 75% according to researches regarding dental caries in other African countries, as there was no previous study in São Tomé and Príncipe [7, 9, 18]); the level of confidence is 95% (*z* = 1.96); *e* is the degree of precision (*e* = 5%); the non-response rate was 5%. Taking α = 5%, β = 20%, and 80% power, the sample size is 304 students in each district and 1824 in total.

Schools were selected in each district, and all sixth-grade students of selected schools were included, amounting to 2,208 students, out of which 278 children were unable to reach. Furthermore, according to the exclusion criteria, 75 unresponsive students were excluded, bringing down the final sample size to 1855.

### Data collection and quality assurance

The research team consisted of the 16th Chinese medical team to São Tomé and Príncipe and staff from local government. Before initiating the survey, all examiners, recorders, and questionnaire interpreters underwent theoretical and operational training under the guidance of a standard examiner from West China Hospital of Stomatology of Sichuan University. The training included a detailed description of dental decay and photographs of teeth with different severity degrees of caries, in accordance with the various codes of the CAST instrument. To assess the reproducibility and consistency of the inter-examiner, each examiner was calibrated by evaluating 18 students, with the Kappa coefficient over 0.85, which was considered substantial. During the course of the survey, 5% of the subjects were randomly re-examined for duplicate examinations to maintain the quality and consistency of data.

A WHO adopted questionnaire (Additional file 6) translated into the local language (Portuguese) and a survey form (Additional file 7) to record the carious status of teeth were applied for data collection, along with written consent.

### Clinical examination

Each student received a clinical examination according to the methods and criteria of the CAST instrument [18] by using a disposable dental mirror and a Community Periodontal Index (CPI) probe under artificial light, which the WHO recommended for epidemiological surveys. The survey form consists of scores of each tooth and general information, including name, gender, date of birth, school, class, study number. Two trained and calibrated examiners carried clinical examinations, two recorders recording the results, and two local staff translating and guiding.

The severity of dental caries was obtained through the use of CAST instrument, ranging from ‘no carious lesion’, to ‘sealed’ (pit and fissure sealant), ‘restored’, ‘enamel caries’, ‘dentine caries’, ‘pulp involvement and abscess/fistula’, ‘teeth lost due to caries’ and ‘others’ (Table 1). Furthermore, the CAST instrument can be converted into DMFT/DMFS (decayed, missing, filled teeth/ surface) data of WHO standard.

**Table 1:** The Caries Assessment Spectrum and Treatment (CAST) instrument codes and descriptions [20]

Characteristic	Code	Description
Sound	0	No visible evidence of a distinct carious lesion
Sealed	1	Pits and/or fissures are at least partially covered with a sealant material
Restored	2	A cavity has been restored with (in) direct restorative material and currently without a carious lesion
Enamel	3	Distinct visual change in enamel only. A clear caries-related discoloration is visible, with or without localized enamel breakdown
Dentine	4	Internal caries-related discoloration in dentine. The lesion is visible through enamel, which may or may not exhibit a visible localized breakdown of enamel
	5	Distinct cavitation into dentine without pulpal involvement
Pulp	6	Involvement of pulp chamber. Distinct cavitation reaching the pulp chamber or only root fragments is present
	7	A pus containing swelling or a pus releasing sinus tract related to the tooth with pulpal involvement due to dental caries is present
Lost	8	The tooth has been removed due to dental caries
Others	9	Does not match with any of the other descriptions

### Questionnaire survey

All students were required to complete a questionnaire under the direction of questionnaire interpreters. The questionnaire was composed of four parts:

1. Family background (single child or not, place of residence)
2. Oral health behavior (tooth brushing frequency, method, and duration, fluoride toothpaste usage)
3. Dietary habits (consumption of sugar, carbonated drinks, candy/chocolate)
4. Self-assessment of oral health, dental knowledge, and attitude (the importance of oral health in the quality of life, the most effective way of preventing dental caries, the necessity of regular dental inspection) (Additional file 6)

### Statistical analysis

All data were entered and statistically analyzed using IBM SPSS Statistics software Version 25.0. Independent sample t-test and Chi-square test were performed to assess the different prevalence of dental caries among different groups and evaluate the relationship between dental caries and related variables. Logistic regression analysis was applied to investigate the association between dental caries prevalence and its risk factors. The results were expressed in mean ± SD, and *P* < 0.05 was considered to be statistically significant in all tests.

## Results

### Sociodemographic characteristics

A total of 1855 students aged 9 to 15 years old agreed to participate in this epidemiological survey, with a response rate of 96.11% among the subjects. These sixth-grade students came from 62 classes out of 10 schools in 6 districts (Table 2). The mean age of the children was 12.23 ± 1.13 years old, most of which 1743 (93.95%) were aged 11–14. About 906 (48.87%) of the respondents were female. 744 (40.13%) participants were from urban areas, 528 (28.48%) were from rural areas, and the rest 583 (31.39%) were from other areas (Table 3).

**Table 2:** Table of district, school and class composition

District	School	Class	N	%
Água Grand	3	26	814	43.88
Mé-Zóchi	1	9	290	15.63
Cantagalo	1	9	189	10.19
Lobata	2	8	240	12.94
Lembá	1	8	213	11.48
Caué	2	4	109	5.88
Total	10	62	1855

**Table 3:** Prevalence of caries, DMFT, DMFS, filling rate and pit and fissure sealant rate of first permanent molar in different groups

Variables	N (%)		Prevalence (%)	DMFT (mean ± SD)	DMFS (mean ± SD)	Filling (%)	PFS (%)
*Sex*							
Male	949	51.13	69.65	1.805 ± 1.474	3.569 ± 3.869	8.76***	3.27**
Female	906	48.87	67.88	1.694 ± 1.552	3.518 ± 4.018	2.76	1.10
*Age*							
≤ 10	27	1.46	77.78	1.750 ± 1.323	3.714 ± 3.568	0	0
11	497	26.79	67.61	1.511 ± 1.541	2.867 ± 3.564	5.13	2.87
12	723	38.96	72.27	1.628 ± 1.509	3.310 ± 3.908	5.74	1.91
13	355	19.14	73.09	1.827 ± 1.521	3.912 ± 4.271	4.82	2.55
14	168	9.06	68.24	1.594 ± 1.501	3.518 ± 3.941	7.06	1.18
≥ 15	85	4.58	65.12	1.581 ± 1.553	3.497 ± 4.573	6.98	2.33
*Residency*							
Urban	744	40.13	73.11	1.624 ± 1.536	3.395 ± 4.091	6.59	1.61
Rural	528	28.48	68.94	1.633 ± 1.484	3.398 ± 3.869	4.73	2.08
Others	583	31.39	69.51	1.612 ± 1.536	3.216 ± 3.801	4.70	3.31
*Single child*							
Yes	136	7.33	61.76	1.221 ± 1.381	2.471 ± 3.313	7.35	2.21
No	1719	92.67	69.34	1.659 ± 1.524	3.405 ± 3.978	5.37	2.22

**P < 0.01, ***P < 0.001: Male compared with female

### Prevalence of dental caries of first permanent molar

The prevalence of dental caries of first permanent molar was 1276 (68.79%), and the mean DMFT and DMFS were 1.751 ± 1.514 and 3.542 ± 3.941 among the participants, respectively. The prevalence was slightly lower in females (67.88%) than in males (69.65%), but the difference was not statistically significant. Also, there was no statistically significant difference between the age groups. Among different residency groups, the prevalence of dental caries of first permanent molar was higher in the urban group (73.11%) than in the rural group (68.94%) as well as in the other groups (69.51%), without significant difference. The rate of caries in the first permanent molar was higher in non-single children (69.34%) than in single children (61.76%), but the difference was not statistically significant (Table 3).

### Filling and pit and fissure sealant rate of first permanent molar

Among all the 1855 participants, the caries filling rate of the first permanent molar was 102 (5.50%), which was lower in females (2.76%) than in males (8.76%), and the difference was statistically significant (*P* < 0.001). But the differences between age groups, residency groups and single child or not groups were not statistically significant. The pit and fissure sealant rate of the first permanent molar was 41 (2.21%), which was also lower in females (1.10%) than in males (3.27%), with a statistically significant difference (*P* < 0.01). Among the age groups, residency groups, and single child or not groups, the difference was not statistically significant (Table 3).

### The distribution of dental caries in different locations and surfaces among first permanent molar

In this study, dental caries of the first permanent molar occurred in mandibular was 1161 (62.59%), and 853 (45.98%) in maxillary, and the difference was statistically significant (*P* < 0.001). Caries occurred on occlusal surface caries was 1237 (66.68%), which was significantly higher than that of other surfaces including mesial, distal, buccal, and lingual surface (390, 21.02%; 584, 31.48%; 609, 32.83%; 605 32.61%; respectively) (*P* < 0.001) (Additional file 1: Table S1).

### Prevalence of CAST codes of first permanent molar

In Additional file 2: Table S2, the results of prevalence of the CAST codes of first permanent molar among sixth-grade children showed that the number of students suffering from enamel caries (code 3) was 1148, with a prevalence of 61.89%, dentin carious lesions (codes 4–5) (864, 46.58%), pulp lesions (codes 6–7) (313, 16.87%), and loss of teeth due to caries (code 8) (60, 3.23%). Moreover, the prevalence of enamel caries has the highest frequency of occurrence among all kinds of lesions with different CAST codes and is statistically significant (*P* < 0.001) (Additional file 2: Table S2).

### The overall prevalence of dental caries in permanent teeth

Among all permanent teeth, the prevalence of dental caries was 1410 (76.01%) and DMFT was 2.753 ± 4.569. The prevalence of dental caries excluding the first permanent molar was 901 (48.57%), and the difference was statistically significant compared to that of the first permanent molar (1276, 68.79%) (*P* < 0.001).

The prevalence of dental caries in mandibular first molar (54.18% in the left and 51.16% in the right) was remarkably higher than that in the maxilla (33.05% in the left and 36.71% in the right), the same to mandibular second molar (24.74% in the left mandible and 27.17% in the right mandible vs. 6.58% in the left maxilla and 9.65% in the right maxilla), with the difference statistically significant (*P* < 0.001). The remaining dental positions with caries in permanent teeth were shown in Additional file 3: Table S3.

### Risk factors associated with dental caries of first permanent molar

According to the questionnaire survey, approximately one-third of all the respondents (32.61%) started brushing teeth at the age of 2 years old, 27.92% by 3 years, 19.03% by 1 year, 10.51% by 4 years, 6.36% by 5 years, and 3.56% by 6 years and above.

From the oral health behavior survey results, 1562 (84.20%) of the subjects had the habit of brushing their teeth every day, and 980 (52.83%) brushed their teeth more than once a day. Those who brushed teeth more than once a day suffered significantly lower prevalence of dental caries of first permanent molar (67.76%) than those who brushed only once a day (71.99%), those who brushed less than once a day (72.62%), and those who brushed occasionally (72.00%), with the difference statistically significant (*P* < 0.05). In addition, there was no significant effect of brushing method, duration of tooth brushing, frequency of toothbrush replacement, and reminding from parents on the prevalence of dental caries. Only 178 (9.60%) students correctly brushed their teeth. Moreover, 1187 (63.99%) of the respondents replaced their toothbrushes every three months, which is undoubtedly the healthy and proper way (Table 4).

**Table 4:** Prevalence of dental caries of first permanent molar associated risk factors according to the questionnaire

Variables	Subjects	N	Prevalence (%)	Chi-square value	*P* value
*Oral health behavior*					
Frequency of tooth brushing*					
Occasionally	125	90	72.00	8.565	0.036
< once a day	168	122	72.62		
Once a day	582	419	71.99		
> once a day	980	645	65.82		
Tooth brushing method					
Horizontally	392	271	69.13	2.281	0.516
Vertically	178	121	67.98		
Mixed	959	649	67.67		
Casually	326	235	72.09		
Duration of tooth brushing					
≤ 1 min	167	118	70.66	1.404	0.705
1–2 min	528	368	69.70		
2–3 min	342	227	66.37		
≥ 3 min	818	563	68.83		
Frequency of toothbrush replacement					
≤ 3 months	1187	791	66.64	7.614	0.055
3–6 months	297	218	73.40		
6–12 months	163	120	73.62		
≥ 1 year	208	147	70.67		
Reminding from parents					
Never	596	424	71.14	3.364	0.186
Occasionally	509	336	66.01		
Usually	750	516	68.80		
*Dietary habits*					
Snacks before bed					
Never	824	557	67.60	1.225	0.747
Occasionally	207	145	70.05		
Sometimes	735	510	69.39		
Usually	89	64	71.91		
Frequency of carbonated drinks					
Never	1091	732	67.09	6.611	0.085
< once a day	375	256	68.27		
Once a day	202	148	73.27		
≥ twice a day	187	140	74.87		
Frequency of desserts*					
Never	355	225	63.38	9.067	0.028
< once a day	662	456	68.88		
Once a day	486	335	68.93		
≥ twice a day	352	260	73.86		
Frequency of candy/chocolate*					
Never	384	248	64.58	8.706	0.033
< once a day	463	313	67.60		
Once a day	441	300	68.03		
≥ twice a day	567	415	73.19		
*Self-assessment of dental health, dental knowledge and attitude*					
Tooth status self-assessment***					
Correct	367	212	57.77	33.918	< 0.001
Incorrect	808	603	74.63		
Unclear	680	461	67.79		
The importance of oral health in the quality of life*					
Agree	873	594	68.04	10.994	0.012
Disagree	252	155	61.51		
Never thought	253	178	70.36		
Unaware	477	349	73.17		
Brushing teeth prevent caries*					
Agree	730	463	63.42	4.863	0.027
Disagree	1190	813	68.32		
Pit and fissure sealant					
Done	252	170	67.46	0.499	0.779
Not done	799	556	69.59		
Never heard	804	550	68.41		
Fluoride for tooth protection					
Used	305	213	69.84		
Never used	417	289	69.30		
Never heard	871	592	67.97	0.537	0.911
Unclear	262	182	69.47		
The necessity of regular dental inspection					
Agree	1429	977	68.37	1.197	0.550
Disagree	140	102	72.86		
Unclear	286	197	68.88		

*P < 0.05, ***P < 0.001: within each variable groups compared

Dietary habits survey showed that 1309 (70.57%) of the subjects liked to eat cake, followed by cookies 1250 (67.39%), chocolate 1152 (62.01%), candy 961 (51.81%), chewing gum 657 (35.42%) and carbonated drinks 331 (17.84%), only 131 (7.06%) dislike none of them (Additional file 4: Figure S1). Almost half of the students, 824 (44.42%), never eat snacks before bed. Most of the respondents, 1091 (58.81%), did not have the habit of drinking carbonated beverages, only 355 (19.14%) did not have the habit of eating dessert, and 1008 (54.34%) ate candy/chocolate once or more per day and 567 (30.57%) twice or more per day. Dessert consumption more than twice a day (prevalence of dental caries 73.86%) and candy/chocolate consumption more than twice a day (prevalence of dental caries 73.19%) were more likely to develop caries, and the difference between each group was statistically significant (*P* < 0.05) (Table 4).

As the results of the self-assessment of oral health, dental knowledge, and attitude survey indicated, only 367 (19.78%) of the respondents had correct self-assessment about dental health, 808 (43.56%) had poor self-assessment, and 680 (36.66%) were unclear about their tooth status. 873 (47.06%) subjects agreed that oral health is essential for health and life. Most of the respondents, 1190 (64.15%), approved brushing teeth can prevent dental caries. Remarkably few 252 (13.58%) claimed to undergo pit and fissure sealant, and 305 (16.44%) used fluoride toothpaste previously. The vast majority of respondents, 1429 (77.04%), agreed that regular dental inspection is necessary. Table 4 also showed that incorrect self-assessment oral health, unaware of dental knowledge, suffered more severe dental caries of first permanent molar (*P* < 0.05).

### Multivariable logistic regression analysis of the risk factors associated with the prevalence of dental caries in first permanent molar

As Table 5 demonstrated, six risk factors were displayed in the final results: frequency of tooth brushing, frequency of desserts eaten, frequency of candy/chocolate eaten, tooth status self-assessment, dental knowledge about the importance of oral health in the quality of life, and brushing teeth prevent caries, among which frequency of candy/chocolate eaten more than twice per day and incorrect tooth status self-assessment were the most critical risk factors for dental caries in the first permanent molar, with OR values of 1.095 and 1.354, respectively (*P* < 0.05).

**Table 5:** Logistic regression analysis of the risk factors associated with the prevalence of dental caries in first permanent molar

Variables	B	SE	Wald	P	OR
Frequency of tooth brushing	0.033	0.057	0.329	0.566	1.033
Frequency of desserts	− 0.100	0.051	3.812	0.051	0.905
Frequency of candy/chocolate*	0.090	0.045	3.950	0.047	1.095
Self-assessment of dental health***	0.303	0.075	16.419	< 0.001	1.354
The importance of oral health in life	0.017	0.035	0.233	0.629	1.017
Brushing teeth prevent caries	− 0.184	0.107	2.924	0.087	0.832

B—regression coefficient; SE—standard error; Wald—a Chi-square value; P—significant level; OR—odds ratios

*P < 0.05; ***P < 0.001

## Discussion

This study is the first epidemiological investigation of dental caries in São Tomé and Príncipe, which could reveal the permanent dentition caries distribution of this country to a great extent. It is of great significance for the prevention and treatment of local dental caries, and can promote the overall oral health situation in São Tomé and Príncipe to develop better.

Our data showed that the prevalence of dental caries of first permanent molar among sixth-grade students in in São Tomé Island (68.79%) and overall prevalence of dental caries (76.01%) were relatively higher than that in some other African countries such as Sudan (57.8%), Tanzania (30.7%), Ethiopia (46.9%), Nigeria (12.2%) and South Africa (36.91%), but lower than that in Eritrea (78%) and Egypt (74%) [9, 18, 21–24]. All these differences might be caused by sociodemographic differences, different sample sizes, or statistical methods. In this study, the mean DMFT (1.751) was significantly higher compared to study done in Ethiopia (1.28) [9], Egypt (1.04) [21], and Nigeria (0.2) [24], and lower compared to a study done in Eritrea (2.5) [18]. According to WHO statistics, the average DT (decayed teeth, DT) score of 12 years old children in the world was 1.86, among which 1.2 in the USA, 1.4 in Japan, 1.8 in Korea, and 0.86 in China, the score of 1.2–2.6 was considered to be a low level of carious prevalence [2, 3]. In São Tomé, this score was higher than most countries and regions globally and higher than the average level in Africa. Furthermore, this might be due to poor oral hygiene practices and dietary habits caused by relatively underdeveloped regional economies and insufficient medical resources.

Using sealants and restorative materials is an effective strategy to increase tooth resistance to caries. As shown in this study, the caries filling rate (5.50%) and pit and fissure sealant rate (2.21%) of the first permanent molar were far behind the global average level [25]. These two indexes were much higher in males than in females, which means males were more active in the treatment and prevention of caries, or females were relatively undervalued. According to Petersen et al. [3], the reason could be due to parents’ unawareness of oral health, lower education level, and relatively low-income family income; it is also possible that children are afraid of dental treatment, thus concealing the symptoms or not cooperating with the dentist. Researches have demonstrated that pit and fissure sealant can effectively prevent caries [26] and is closely related to the prevalence of caries among children aged 6–17 years old [27]. This survey showed that the caries filling rate and pit and fissure sealant rate in São Tomé Island were both at a low level, indicating that strengthening oral health education, implementing oral health promotion to schools, improving oral health care knowledge, and popularizing caries preventing measures are necessary.

This study found that the prevalence of dental caries of mandibular first permanent molars (62.59%) was significantly higher than that of the maxilla (45.98%) due to the different positions and anatomical structures. Dental caries most commonly occurred in the occlusal surface, followed by the buccal and lingual surfaces, which is consistent with the study done by Batchelor et al. [28]. In addition, the results showed that 61.89% of the lesions among first permanent molars were enamel caries, which was due to the early eruption of the first permanent molar, deep pits and fissures of the occlusal surface, thinner enamel and dentin, relatively poor degree of mineralization and difficulty to clean, thus leading to the susceptibility to dental caries. This result is consistent with studies conducted in other countries [29, 30].

Brushing can remove dental plaque and maintain good oral hygiene, and brushing teeth twice per day with the appropriate method and fluoride toothpaste is considered the most acceptable and effective in preventing caries [31]. Fluoride prevents dental caries by reducing the solubility of enamel, promoting enamel remineralization, and affecting the metabolism of cariogenic bacteria [32]. In our study, most students started brushing their teeth before school age, only 52.83% of the subjects brushed their teeth twice daily, and merely 16.44% used toothpaste containing fluoride, indicating that oral hygiene education should be strengthened in the promotion of fluoride toothpaste for caries prevention.

Restricting sugars to 5% of energy intake can minimize the risk of dental caries [33]. In this study, more consumption of desserts and candy/chocolate was significantly associated with dental caries. Good oral hygiene behavior can effectively reduce the incidence of caries, while poor dental hygiene behavior conversely. Sugar provides the energy and nutrition for the proliferation of bacteria, which is the most crucial factor leading to caries. Frequent sugar consumption can cause a long-term acidic oral environment and enamel demineralization, thus leading to caries. Hence, the change in dental hygiene behavior and diet can modify or eliminate the causes of dental caries [21, 34].

As demonstrated in this study, poor self-assessment of dental health was associated with a higher prevalence of dental caries, the same to the incorrect self-assessment of dental knowledge and attitude, such as the importance of oral health in the quality of life and brushing teeth to prevent caries. This result is similar to a study conducted in Ethiopia and Nepal [9, 35]. The reason might be that children who did not receive proper oral health education might have got incorrect messages that could contribute to dental caries.

Despite all the risk factors, such as frequency of tooth brushing, desserts, candy/chocolate consumption, self-assessment of dental health and knowledge, the results of logistic regression analysis indicated that frequency of candy/chocolate eaten more than twice per day and incorrect tooth status self-assessment were the most critical risk factors for dental caries in the first permanent molar, with OR values of 1.095 and 1.354, respectively. Those parents, schools, and health administration departments did not have enough publicity in oral health knowledge and ignored the importance of propaganda. Correct self-assessment can effectively reduce dental caries. Therefore, the publicity of oral health knowledge should be increased in the future so that school children can develop good oral health concepts and good oral hygiene habits.

This study also has its limitations. Due to limited time and short-staffed, we obtained detailed CAST scores only for the first permanent molar, and DMFT scores for the rest of permanent teeth. For the questionnaire, some of the participants were not old enough to understand all the questions, so staff from the local government helped explain the questionnaire. Nevertheless, further studies concerning other age groups with more detailed dental carious status and related risk factors are thus called for.

## Conclusions

In conclusion, this study revealed that the prevalence of dental caries was considerably high, which is a common public health problem. The DMFT and DMFS of the first permanent molar were 1.751 ± 1.514 and 3.542 ± 3.941, respectively. Furthermore, these scores are relatively higher compared to studies from other African developing countries. Frequency of candy/chocolate eaten more than twice per day and incorrect tooth status self-assessment were the most critical risk factors for dental caries in the first permanent molar among sixth-grade students in São Tomé Island.

## Supplementary Information

**Additional file 1: Table S1**. Different locations and tooth surfaces and position caries status of first permanent molar

**Additional file 2: Table S2**. Prevalence of CAST codes of first permanent molar among sixth-grade students (highest score per mouth used)

**Additional file 3: Table S3**. Distribution of caries of permanent teeth in different dental position

**Additional file 4: Figure S1**. Favorite foods of sixth-grade students

**Additional file 5**. The list of students of sixth-grade in São Tomé Island (Portuguese)

**Additional file 6**. Questionnaire (Portuguese and English)

**Additional file 7**. CAST instrument of Oral Health Assessment (English)

**Additional file 8**. Informed consent (Portuguese)

**Additional file 9**. Ethics Committee consent (Portuguese)

**Additional file 10**. Authorization from the Ministry of Education (Portuguese)

**Additional file 11**. Official letter of authorization from the Ministry of Education (Portuguese)

## Data Availability

The complete data set supporting the conclusions of this article is available from the corresponding author and can be accessed upon reasonable request.

## Abbreviations

DMFT: Decayed, missing, filled teeth
DMFS: Decayed, missing, filled surface
WHO: World Health Organization
CAST: Caries Assessment Spectrum and Treatment
PFS: Pit and fissure sealant
SPSS: Statistical Package for Social Science
