# Supplementary material for: Prevalence of dental caries in the first permanent molar and associated risk factors among sixth-grade students in São Tomé Island
Source: BMC Oral Health. 2021 Sep 28;21:483. doi: 10.1186/s12903-021-01846-z (PMC8479893; doi:10.1186/s12903-021-01846-z)
Supplement: Supplementary file 1 — Additional file 1: Table S1. Different locations and tooth surfaces and position caries status of first permanent molar [file 12903_2021_1846_MOESM1_ESM.docx]

**Additional file 1:**

**Table S1** Different locations and tooth surfaces and position caries status of first permanent molar

|  |  | N | Prevalence  (%) | Chi-square value | P-value |
| --- | --- | --- | --- | --- | --- |
| Dentition | Maxillary | 853 | 45.98 | 103.036 | <0.001 |
|  | Mandibular | 1161 | 62.59*** |  |  |
| Surface | Mesial | 390 | 21.02 |  |  |
|  | Occlusal | 1237 | 66.68*** | 886.605 | <0.001 |
|  | Distal | 584 | 31.48 |  |  |
|  | Buccal | 609 | 32.83 |  |  |
|  | Lingual | 605 | 32.61 |  |  |

*** P<0.001: Mandibular dentition compared with maxillary dentition; Occlusal surface compared with other surfaces.
