# Supplementary material for: Prevalence of dental caries in the first permanent molar and associated risk factors among sixth-grade students in São Tomé Island
Source: BMC Oral Health. 2021 Sep 28;21:483. doi: 10.1186/s12903-021-01846-z (PMC8479893; doi:10.1186/s12903-021-01846-z)
Supplement: Supplementary file 2 — Additional file 2: Table S2. Prevalence of CAST codes of first permanent molar among sixth-grade students (highest score per mouth used) [file 12903_2021_1846_MOESM2_ESM.docx]

**Additional file 2:**

**Table S2** Prevalence of CAST codes of first permanent molar among sixth-grade students (highest score per mouth used)

| Lesions | CAST code | N | Prevalence  (%) | Chi-square value | P-value |
| --- | --- | --- | --- | --- | --- |
| Enamel | 3 | 1148 | 61.89*** | 1838.646 | <0.001 |
| Dentin | 4-5 | 864 | 46.58 |  |  |
| Pulp | 6-7 | 313 | 16.87 |  |  |
| Lost | 8 | 60 | 3.23 |  |  |

*** P<0.001: Enamel caries compared with detin, pulp carious lesions and tooth lost.
