# Supplementary material for: Prevalence of dental caries in the first permanent molar and associated risk factors among sixth-grade students in São Tomé Island
Source: BMC Oral Health. 2021 Sep 28;21:483. doi: 10.1186/s12903-021-01846-z (PMC8479893; doi:10.1186/s12903-021-01846-z)
Supplement: Supplementary file 3 — Additional file 3: Table S3. Distribution of caries of permanent teeth in different dental position [file 12903_2021_1846_MOESM3_ESM.docx]

**Additional file 3:**

**Table S3** Distribution of caries of permanent teeth in different dental position

| Dental  position | Right maxilla | Prevalence  (%) | Left maxilla | Prevalence  (%) | Left mandible | Prevalence  (%) | Right mandible | Prevalence  (%) |
| --- | --- | --- | --- | --- | --- | --- | --- | --- |
| Central incisor | 16 | 0.86 | 12 | 0.65 | 0 |  | 0 |  |
| Lateral incisor | 18 | 0.97 | 10 | 0.54 | 0 |  | 0 |  |
| Canine | 6 | 0.32 | 0 |  | 4 | 0.22 | 0 |  |
| 1^st^ premolar | 68 | 3.67 | 72 | 3.88 | 20 | 1.08 | 16 | 0.86 |
| 2^nd^ premolar | 90 | 4.85 | 87 | 4.69 | 95 | 5.12 | 75 | 4.04 |
| 1^st^ molar | 681 | 36.71 | 613 | 33.05 | 1005 | 54.18*** | 949 | 51.16*** |
| 2^nd^ molar | 179 | 9.65 | 122 | 6.58 | 459 | 24.74*** | 504 | 27.17*** |
| 3^rd^ molar | 0 |  | 0 |  | 0 |  | 1 | 0.05 |

*** P<0.001: Mandibular molars compared with maxillary molars that in the same position.
