# Supplementary material for: Prevalence of dental caries in the first permanent molar and associated risk factors among sixth-grade students in São Tomé Island
Source: BMC Oral Health. 2021 Sep 28;21:483. doi: 10.1186/s12903-021-01846-z (PMC8479893; doi:10.1186/s12903-021-01846-z)
Supplement: Supplementary file 4 — Additional file 4: Figure S1. Favorite foods of sixth-grade students [file 12903_2021_1846_MOESM4_ESM.docx]

**Additional file 4:**

**Figure S1** Favorite foods of sixth-grade students
