# Supplementary material for: Prevalence of dental caries in the first permanent molar and associated risk factors among sixth-grade students in São Tomé Island
Source: BMC Oral Health. 2021 Sep 28;21:483. doi: 10.1186/s12903-021-01846-z (PMC8479893; doi:10.1186/s12903-021-01846-z)
Supplement: Supplementary file 5 — Additional file 5. The list of students of sixth-grade in São Tomé Island (Portuguese) [file 12903_2021_1846_MOESM5_ESM.docx]

REPÚBLICA DEMOCRÁTICA***
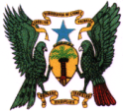
*** DE S.TOMÉ E PRÍNCIPE

(Unidade-Disciplina-Trabalho)

**Ministério da Educação e Ensino Superior**

**Direcção do Ensino Básico**

**Dados dos Alunos da 6ª Classe das Escolas Básicas**

Direcção do Ensino Básico, em S.Tomé, 26 de Janeiro de 2021

A Directora

______________________________________

Deolinda Carvalho
