# Supplementary material for: Prevalence of dental caries in the first permanent molar and associated risk factors among sixth-grade students in São Tomé Island
Source: BMC Oral Health. 2021 Sep 28;21:483. doi: 10.1186/s12903-021-01846-z (PMC8479893; doi:10.1186/s12903-021-01846-z)
Supplement: Supplementary file 6 — Additional file 6. Questionnaire (Portuguese and English) [file 12903_2021_1846_MOESM6_ESM.pdf]

Questionário sobre prevalência de cárie nos primeiros molares permanentes de crianças em São Tomé e Príncipe

Data: 2021 -- --

Escola： Turma： Nº：

Nome： Sexo： Idade： Data de nascimento：

1. Você escova seus dentes? ( )

A.Sim B. Não

2. Com que idade você começou a escovar os dentes? ( ) anos.

3. Quantas vezes você escova os dentes por dia? ( )

A. escovar quando apetece B. nem escovar todos os dias

C.uma vez por dia D. mais de 2 vezes ao dia

4. Qual método você usa para escovar os dentes? ( )

A. escovar na horizontal B.escovar na veritical

C. escovar na horizontal e veritical D. sem método de fixação

5. Por quanto tempo você costuma escovar os dentes? ( )

A. menos de um minuto B. 1 ou 2 minutos C.2-3 minutos D. mais de 3 minutos

6. Com que frequência você troca a escova de dentes que usa? ( )

A. menos de 3 meses B. 3-6 meses C. 6 meses-1 ano D. 1 ano ou mais

7. Seus pais vão lembrá-lo de escovar os dentes todos os dias? ( )

A. Não B. de vez em quando C. Frequentemente

8. Você come doces antes de ir para a cama? ( )

A. Nunca B. ao calhar C. Às vezes D. Frequentemente

9. Você gosta dos seguintes alimentos? (Múltipla escolha)

☐ bebidas carbonatadas ☐ bolacha ☐ Não gosto de tudo

☐ doce ☐ bolo

☐ pastilha com açucar ☐ chocolate

10. Com que frequência você come os seguintes alimentos?

	nunca	menos de uma vez por dia	uma vez por dia	2 vezes ou mais por dia
bebidas carbonatadas(cola)	☐	☐	☐	☐
Sobremesa(bolo)	☐	☐	☐	☐
Chocolate/doces etc.	☐	☐	☐	☐

11. Quantos dentes cariados você tem? ( )

A. Nenhum B. 1 dente C. 2-3 dentes D. mais de 3 dentes E. Não sei

12. Você acha que a cárie dentária tem provocado muito prejuízo na nossa saúde e na nossa vida? ( )

A.Sim B. Não C. nunca pensei nisso D. não sei

13. Qual forma você acha que é mais eficaz de prevenir a cárie dentária? ( )

A. Não coma antes de dormir B. Comer menos doces

C. Usar creme dental com flúor D. Escovar os dentes de manhã e à noite

14. Você já fez o tratamento de fechamento de fossas e fissuras? ( )

A.Sim B. Não C. Não sei o que isso.

15. Você já ouviu falar de creme dental com flúor? Já usou? ( )

A. Não ouvi falar B. Ouvi falar mas nunca usei C. Usei D. Não sei

16. Você acha que precisamos verificar os dentes regularmente? ( )

A. Sim B. Não precisa C. Não tenho certeza / não sei

17. Você é filho único da sua família? ( )

A. Sim B. Não

18. Onde você mora? ( )

A. Urbano B. Rural C. Outro

Questionnaire on the prevalence of dental caries in first permanent molars of students in São Tomé and Príncipe

Date: 2021-   -

School: Grade: Class: No:

Name: Gender: Age: Date of birth:

1. Do you brush your teeth? ( )

A. Yes   B. No

2. When did you start brushing your teeth? Since ( ) years old.

3. How many times do you brush your teeth per day? ( )

A. Occasionally B. Sometimes C. once a day D.≥twice a day

4. How do you use to brush your teeth? ( )

A. Horizontally B. Vertically C. Both horizontally and vertically

D. Randomly

5. How long do you brush your teeth? ( )

A. < 1 minute B. 1- 2 minutes C. 2-3 minutes D. > 3 minutes

6. How often do you replace your toothbrush? ( )

A.≤3 months B. 3-6 months C. 6 months-1 year D. > 1 year

7. Will your parents remind you to brush your teeth every day? ( )

A. No B. Occasionally C. Often

8. Do you eat sweets before going to bed? ( )

A. Never B. Occasionally C. Sometimes D. Often

9. Do you like the following foods?

☐ carbonated drinks   ☐ cookie   ☐ none of them

☐ sweet   ☐ cake

☐ sugar gum   ☐ chocolate

10. How often do you eat the following foods?

	Never	< once a day	once a day	⩾twice a day
carbonated drinks (cola)	☐	☐	☐	☐
Dessert (cake)	☐	☐	☐	☐
Chocolate/sweets etc.	☐	☐	☐	☐

11. How many decayed teeth do you have? ( )

A. none B. 1 tooth C. 2-3 teeth D. ⩾3: ( ) teeth E. I don't know

12. Do you think that tooth decay has a great impact on our general health and daily life? ( )

A. Yes B. No C. Never thought about it D. I don't know

13. Which way do you think is the most effective way to prevent tooth decay? ( )

A. Don't eat before bedtime B. Eat less sweets

C. Use fluoride toothpaste D. Brush your teeth twice a day

14. Have you ever done the pit and fissure sealant? ( )

A. Yes B. No C. I don't know what is pit and fissure sealant

15. Have you ever heard of fluoride toothpaste? Have you used it? ( )

A. Never heard of it B. I've heard of it but never used it

C. I have used it D. I don't know

16. Do you think it's necessary to do the dental inspection regularly? ( )

A. Yes B. No C. Not sure / I don't know

17. Are you the only child in your family? ( )

A. Yes B. No

18. Where do you live? ( )

A. Urban B. Rural C. Others
