# Supplementary material for: Prevalence of dental caries in the first permanent molar and associated risk factors among sixth-grade students in São Tomé Island
Source: BMC Oral Health. 2021 Sep 28;21:483. doi: 10.1186/s12903-021-01846-z (PMC8479893; doi:10.1186/s12903-021-01846-z)
Supplement: Supplementary file 7 — Additional file 7. CAST instrument of Oral Health Assessment (English) [file 12903_2021_1846_MOESM7_ESM.pdf]

Records of caries in the first permanent molars

Data of examination: ____–____–____

School:	Class:	Study Number:	
Name:	Gender:	Age:	Date of Birth:

	Mesial ( )			Mesial ( )	
Buccal ( )	Occlusal ( )	Lingual ( )	Lingual ( )	Occlusal ( )	Buccal ( )
	Distal ( )			Distal ( )	

tooth site: 16	tooth site: 26
tooth site: 46	tooth site: 36

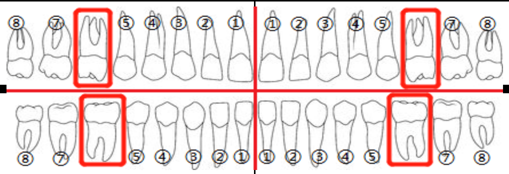

	Distal ( )			Distal ( )	
Buccal ( )	Occlusal ( )	Lingual ( )	Lingual ( )	Occlusal ( )	Buccal ( )
	Mesial ( )			Mesial ( )	

CAST coding:

(Score 0) Sound;
(Score 1) Sealed;
(Score 2) Restored without carious lesions;
(Score 3) Enamel caries;
(Score 4) Shadows of dentin;
(Score 5) Dentine caries;
(Score 6) Endodontic lesions;
(Score 7) Swelling/fistula;
(Score 8) Tooth removed because of dental caries;
(Score 9) Others.
