# Supplementary material for: Prevalence of dental caries in the first permanent molar and associated risk factors among sixth-grade students in São Tomé Island
Source: BMC Oral Health. 2021 Sep 28;21:483. doi: 10.1186/s12903-021-01846-z (PMC8479893; doi:10.1186/s12903-021-01846-z)
Supplement: Supplementary file 8 — Additional file 8. Informed consent (Portuguese) [file 12903_2021_1846_MOESM8_ESM.pdf]

CONSENTIMENTO INFORMADO PARA PARTICIPAÇÃO NO ESTUDO:

Caro pai/encarregado de educação,

Vimos por este meio solicitar a autorização para a participação do seu filho(a)/educando(a) no estudo "investigação epidemiológica de carie dentária do grupo de crianças de 6ª classe em São Tomé " em que se pretende realizar os seguintes procedimentos:

- inspeção bucal para avaliação da cárie dos primeiros molares permanentes, incluindo a taxa de cárie, DMFS (superfície deteriorada, ausente e preenchida) e a taxa de selante de fossas e fissuras.
- aplicação de espuma de flúor para prevenir a cárie dentária no futuro.
- aplicação de um questionário com a finalidade de colher informações acerca de fatores relacionados à cárie, como hábitos alimentares, fatores socioeconômicos, ingestão de flúor, influência familiar, etc., incluindo especificamente: gênero, distribuição urbana e rural, frequência de escovar os dentes, frequência de comer sobremesa, comer sobremesa / beber leite antes de dormir, etc.

A participação neste estudo vai ajudar a nossa equipa a conhecer a situação da doença de cárie dentária em STP, adquirindo os dados para análise dos fatores riscos que causam cárie, fornecendo base dos dados para a implementação de medidas direcionadas de prevenção e tratamento da cárie bucal; pode efetivamente orientar os cuidados de saúde preventiva bucal no país e promover a saúde bucal geral de São Tomé e Príncipe em uma boa direção. Os procedimentos não são dolorosos nem implicam desconforto para o seu filho(a)/educando(a). **A participação neste estudo é livre e voluntária e a não participação no estudo não trará nenhum inconveniente e/ou sanção ao seu filho(a)/educando(a).**

Declaro que tomei conhecimento do estudo que está a ser realizado na escola do meu filho(a)/educando(a) pela equipa médica chinesa e que estou esclarecido quanto ao mesmo.

Dessa forma, Eu ____, maior, de _________ anos de idade, autorizo a participação do meu filho(a)/educando(a) ______________________________(Nome do filho) a participar neste estudo.

INFORMED CONSENT FOR PARTICIPATION IN THE STUDY:

Dear parent/guardian,

We hereby request permission for your child/children to participate in the study "Epidemiological Investigation of Dental Caries in the Group of Children in the 6th Grade in São Tomé":

- oral inspection for caries assessment of first permanent molars, including caries rate, DMFS (decayed, missing and filled surface) and pit and fissure sealant rate.
- application of fluoride foam to prevent tooth decay in the future.
- application of a questionnaire with the purpose of collecting information about caries-related factors such as eating habits, socioeconomic factors, fluoride intake, family influence, etc., including specifically: gender, urban and rural distribution, frequency of brushing teeth, frequency of eating dessert, eating dessert/drinking milk before bedtime, etc.

Participation in this study will help our team to know the situation of dental caries disease in STP, acquiring the data for analysis of the risk factors that cause caries, providing data basis for the implementation of targeted measures of prevention and treatment of oral caries; it can effectively guide the preventive oral health care in the country and promote the general oral health of Sao Tome and Principe in a good direction. The procedures are not painful or uncomfortable for your child/children. **Participation in this study is free and voluntary and non-participation in the study will not bring any inconvenience and/or sanction to your child/children.**

I declare that I have read about the study that is being conducted at my child/children's school by the Chinese medical team and that I am clear about it.

Thus, I ____, of legal age,________, authorize my child/children____, to participate in this study.
