# Supplementary material for: Prevalence of dental caries in the first permanent molar and associated risk factors among sixth-grade students in São Tomé Island
Source: BMC Oral Health. 2021 Sep 28;21:483. doi: 10.1186/s12903-021-01846-z (PMC8479893; doi:10.1186/s12903-021-01846-z)
Supplement: Supplementary file 9 — Additional file 9. Ethics Committee consent (Portuguese) [file 12903_2021_1846_MOESM9_ESM.pdf]

PARECER COMITÉ DE ÉTICA

PROTOCOLO REF: 012/2020

"Projeto de Investigação Epidemiológica da Cárie Dentária do grupo de crianças de 12 anos de idade em São Tomé e Príncipe"

Parecer acerca da apreciação do protocolo:

Interesse do estudo:

- O estudo tem interesse para ser realizado no território nacional, tendo em conta trazer elementos e informações acerca da epidemiologia da cárie dentária nas crianças, em uma idade chave do seu desenvolvimento. Estas informações permitem direcionar e implementar as ações adequadas para a prevenção da doença bucal e promoção da saúde oral.
- Até ao momento não foi realizado nenhum estudo semelhante em São Tomé e Príncipe.

CHECK-LIST:

a) Título: descritivo e objetivo – **PRESENTE.**

b) Identificação completa do(s) investigador(es) responsável(is) e entidade(s) de origem, quando aplicável. – **PRESENTE.**

c) *Curriculum vitae* do(s) investigador(es) - formato curto – **PRESENTE.**

d) Introdução: justificando o estudo, enunciando a questão clínica que o desencadeou e a respetiva pertinência com base numa revisão bibliográfica adequada. – **PRESENTE.**

e) Metodologia – **CLARA E OBJETIVA.**

f) Recursos / orçamento / protocolo financeiro e origem de eventuais financiamentos. – **NÃO SE APLICA.**

g) Cronograma. - **PRESENTE.**

h)Declaração do responsável da unidade de saúde, relativo à disponibilidade para a realização do estudo, o acordo quanto às condições estruturais e de logísticas para a sua realização nomeadamente no que concerne à equipa de investigação a envolver no estudo. – **NÃO SE APLICA.**

i) Pressupostos Éticos – **CONSENTIMENTO INFORMADO PRESENTE.**

Conclusão: O parecer com base na avaliação realizada **é favorável**, dado o cumprimento das normas estipuladas pelo comité de ética.

CESIC
Comissão de Ética na Saúde
Para Investigação Científica
São Tomé e Príncipe

Comité de Ética de São Tomé e Príncipe

São Tomé e Príncipe, 12 de Janeiro de 2021
