# Supplementary material for: Prevalence of dental caries in the first permanent molar and associated risk factors among sixth-grade students in São Tomé Island
Source: BMC Oral Health. 2021 Sep 28;21:483. doi: 10.1186/s12903-021-01846-z (PMC8479893; doi:10.1186/s12903-021-01846-z)
Supplement: Supplementary file 10 — Additional file 10. Authorization from the Ministry of Education (Portuguese) [file 12903_2021_1846_MOESM10_ESM.pdf]

REPÚBLICA DEMOCRÁTICA DE S.TOMÉ E PRÍNCIPE
(Unidade – Disciplina - Trabalho)
MINISTÉRIO DA EDUCAÇÃO E ENSINO SUPERIOR
Direcção do Planeamento e Inovação Educativa

São Tomé

DECLARAÇÃO DE AUTORIZAÇÃO

Conforme a Vossa solicitação, e com autorização de Sua Excelência a Ministra da Educação e Ensino Superior, **está autorizada à equipa Médica Chinesa Assistente a S. Tomé e Príncipe, a implementação do "Projecto de Investigação Epidemiológica da Cárie Dentária" no grupo de crianças** dos Ensinos Básico e Secundário de São Tomé e Príncipe.

Com os meus melhores Cumprimentos.

Direcção do Planeamento e Inovação Educativa em S. Tomé, 19 Março de 2021.

A Directora

REPÚBLICA DEMOCRÁTICA DE S. TOMÉ E PRÍNCIPE - Ministério da Educação e Ensino Superior - D.P.I.E. - Direcção Planeamento Inovação Educativa

Bleizy da Vera Cruz José da Costa
