# Supplementary material for: Prevalence of dental caries in the first permanent molar and associated risk factors among sixth-grade students in São Tomé Island
Source: BMC Oral Health. 2021 Sep 28;21:483. doi: 10.1186/s12903-021-01846-z (PMC8479893; doi:10.1186/s12903-021-01846-z)
Supplement: Supplementary file 11 — Additional file 11. Official letter of authorization from the Ministry of Education (Portuguese) [file 12903_2021_1846_MOESM11_ESM.pdf]

REPÚBLICA DEMOCRÁTICA DE S.TOMÉ E PRÍNCIPE
(Unidade – Disciplina - Trabalho)
MINISTÉRIO DA EDUCAÇÃO E ENSINO SUPERIOR
Direcção do Planeamento e Inovação Educativa

Exma. Representante da Equipa
Médica Chinesa Assistente a STP
Wang Rui

São Tomé

Ofício Nº 26/DPIE- MEES-2021

A Direcção de Planeamento e Inovação Educativa, no quadro das suas actividades, vem por este meio enviar em anexo a declaração referente a autorização de **implementação do "Projecto de Investigação Epidemiológica da Cárie Dentária" no grupo de crianças** dos Ensinos Básico e Secundário de São Tomé e Príncipe.

Com os meus melhores Cumprimentos.

Direcção do Planeamento e Inovação Educativa em S. Tomé, 23 Março de 2021.

A Directora

____

Bleizy da Vera Cruz José da Costa
